# Supplementary material for: Inferring structure and parameters of stochastic reaction networks with logistic regression
Source: PLoS One. 2026 Feb 12;21(2):e0341639. doi: 10.1371/journal.pone.0341639 (PMC12900436; doi:10.1371/journal.pone.0341639)
Supplement: S4 Tables — Details of the results on logistic regression fitting to the modes discussed in the paper. (PDF) [file pone.0341639.s004.pdf]

S4 Tables

Table S4A. Species identification in the TK model using logistic regression (Case 1). Symbol "+" indicates that estimated coefficients are significant and have positive signs. Symbol ✓ indicates correct identification of the corresponding reaction.

No.	Reaction	A_1	A_2		# of Obs.
1	$A_1 + A_2 \to 2A_1$	+	+	✓	173,174
2	$A_1 + A_2 \to 2A_2$	+	+	✓	172,751
3	$A_1 \to \emptyset$	+		✓	6,535
4	$\emptyset \to A_1$			✓	5,982
5	$A_2 \to \emptyset$		+	✓	5,495
6	$\emptyset \to A_2$ (reference)				6,062

Table S4B. Multinomial logistic regression model fitting summary table for the TK model with symmetric reaction rate (Case 1). Highly significant positive values are shown in bold. Refer also to the histogram of Z-values in Fig S3.

Reactant:Reaction	Estimate	Std. Error	Z-score
A_1:1	1.1882	0.1867	**6.365*****
A_1:2	1.1960	0.1867	**6.406*****
A_1:3	1.3074	0.1461	**8.952*****
A_1:4	0.0456	0.0673	0.678
A_1:5	0.0611	0.0689	0.886
A_2:1	1.1800	0.1608	**7.341*****
A_2:2	1.1801	0.1608	**7.341*****
A_2:3	0.0296	0.0777	0.381
A_2:4	0.0462	0.0673	0.687
A_2:5	1.0224	0.1412	**7.238*****

The intercept terms are omitted. $^{*} < .05$, $^{**} < .01$, and $^{***} < .001$

Table S4C. Species identification in the TK model using logistic regression (Case 2). Symbol "+" indicates that estimated coefficients are significant and have positive signs. Symbols ✓indicates correct identification of the corresponding reaction

No.	Reactions	A_1	A_2		# of Obs.
1	$A_1 + A_2 \to 2A_1$	+	+	✓	367,218
2	$A_1 + A_2 \to 2A_2$	+	+	✓	386,551
3	$A_1 \to \emptyset$	+		✓	1,016
4	$\emptyset \to A_1$			✓	19,918
5	$A_2 \to \emptyset$		+	✓	28,441
6	$\emptyset \to A_2$ (reference)				10,022

Table S4D. Multinomial logistic regression model fitting summary table for the TK model with asymmetric reaction rate (Case 2).

Reactant:Reaction	Estimate	Std. Error	Z-score
A_1:1	1.0359	0.0570	**18.166*****
A_1:2	1.0350	0.0570	**18.152*****
A_1:3	1.1239	0.1497	**7.506*****
A_1:4	0.0116	0.0105	1.104
A_1:5	0.0038	0.0104	0.364
A_2:1	0.9676	0.1217	**7.952*****
A_2:2	0.9604	0.1217	**7.893*****
A_2:3	0.0053	0.0160	0.331
A_2:4	0.0095	0.0108	0.881
A_2:5	0.9316	0.0598	**15.590*****

The intercept terms are omitted. * < .05, ** < .01, and *** < .001

Table S4E. Species identification in the heat shock response model using logistic regression (Case 1). Symbol "+" indicates that estimated coefficients are significant and have positive signs. Symbol ✓ indicates correct identification of the corresponding reaction.

No.	Reactions	P_1	P_2	R_1		# of Obs.
1	$P_1 \to R_1$	+			✓	18,909
2	$P_2 \to R_1$		+		✓	27,364
3	$R_1 \to P_2$			+	✓	35,996
4	$R_1 \to 2R_1$			+	✓	36,322
5	$R_1 + P_2 \to \emptyset$		+	+	✓	10,068
6	$R_1 \to \emptyset$			+	✓	36,679
7	$P_1 \to \emptyset$	+			✓	19,069
8	$P_2 \to \emptyset$		+		✓	27,536
9	$\emptyset \to P_1$, $P_2 \to P_1 + P_2$		+		✓	37,676
10	$\emptyset \to P_2$, $P_1 \to P_1 + P_2$ (reference)					28,758

Table S4F. Multinomial logistic regression model fitting summary table for the Heat Shock Response model for Case 1.

Reactant:Reaction	Estimate	Std. Error	Z-score
P_1:1	0.3805	0.0388	**9.799*****
P_1:2	-0.6841	0.0341	-20.072***
P_1:3	-0.6487	0.0319	-20.325***
P_1:4	-0.6437	0.0319	-20.213***
P_1:5	-0.6521	0.0467	-13.970***
P_1:6	-0.6453	0.0318	-20.306***
P_1:7	0.2892	0.0386	**7.488*****
P_1:8	-0.6993	0.0340	-20.555***
P_1:9	-0.6376	0.0316	-20.144***
P_2:1	-0.1189	0.0466	-2.551*
P_2:2	0.9539	0.0426	**22.397*****
P_2:3	-0.0112	0.0392	-0.286
P_2:4	-0.0424	0.0391	-1.087
P_2:5	0.9381	0.0594	**15.793*****
P_2:6	-0.0491	0.0389	-1.262
P_2:7	-0.0168	0.0466	-0.360
P_2:8	0.9739	0.0425	**22.893*****
P_2:9	0.6749	0.0392	**17.239*****
R_1:1	0.0704	0.0381	1.848
R_1:2	0.0200	0.0344	0.581
R_1:3	1.0581	0.0328	**32.289*****
R_1:4	1.0417	0.0327	**31.052*****
R_1:5	0.9969	0.0489	**20.402*****
R_1:6	1.0139	0.0326	**31.114*****
R_1:7	0.0538	0.0379	1.417
R_1:8	0.0395	0.0344	1.149
R_1:9	0.0399	0.0319	1.251

The intercept terms are omitted. * < .05, ** < .01, and *** < .001

Table S4G. Species identification in the Heat Shock Response model using logistic regression (Case 2a). Symbol "+" indicates that estimated coefficients are significant and have positive signs. Symbol ✓ indicates correct identification of the corresponding reaction.

No.	Reactions	P_1	P_2	R_1		# of Obs.
1	$P_1 \to R_1$					14,793
2	$P_2 \to R_1$		+		✓	18,953
3	$R_1 \to P_2$			+	✓	28,571
4	$R_1 \to 2R_1$			+	✓	28,763
5	$R_1 + P_2 \to \emptyset$		+	+	✓	5,749
6	$R_1 \to \emptyset$			+	✓	28,411
7	$P_1 \to \emptyset$					14,847
8	$P_2 \to \emptyset$		+		✓	18,997
9	$\emptyset \to P_1,\ P_2 \to P_1 + P_2$		+		✓	29,252
10	$\emptyset \to P_2,\ P_1 \to P_1 + P_2$ (reference)					14,805

Table S4H. Multinomial logistic regression model fitting summary table for the Heat Shock Response model for Case 2a.

Reactant:Reaction	Estimate	Std. Error	Z-score
P_1:1	-0.0353	0.0432	-0.817
P_1:2	-1.0505	0.0399	-26.325***
P_1:3	-1.0163	0.0369	-27.532***
P_1:4	-1.0362	0.0369	-28.107***
P_1:5	-1.0136	0.0554	-18.286***
P_1:6	-0.9902	0.0370	-26.791***
P_1:7	-0.0170	0.0432	-0.395
P_1:8	-0.9633	0.0399	-24.115***
P_1:9	-1.0073	0.0369	-27.308***
P_2:1	0.0031	0.0464	0.066
P_2:2	1.0170	0.0448	**22.677***
P_2:3	-0.0048	0.0404	-0.118
P_2:4	0.0183	0.0403	0.455
P_2:5	1.0567	0.0655	**16.139***
P_2:6	-0.0081	0.0404	-0.200
P_2:7	-0.0083	0.0463	-0.180
P_2:8	0.9227	0.0447	**20.634***
P_2:9	0.5895	0.0405	**14.542***
R_1:1	0.1125	0.0392	2.875**
R_1:2	0.0585	0.0371	1.577
R_1:3	1.0653	0.0352	**30.287***
R_1:4	1.0378	0.0351	**29.568***
R_1:5	1.0028	0.0560	**17.908***
R_1:6	1.0533	0.0352	**29.930***
R_1:7	0.0666	0.0390	1.707
R_1:8	0.0802	0.0371	2.165*
R_1:9	0.0833	0.0340	2.452*

The intercept terms are omitted. * < .05, ** < .01, and *** < .001

Table S4I. Species identification in the Heat Shock Response model using logistic regression (Case 2b) with 20 trajectories. Symbol "+" indicates that estimated coefficients are significant and have positive signs. Symbol ✓ indicates correct identification of the corresponding reaction.

No.	Reactions	P_1	P_2	R_1		# of Obs.
1	$P_1 \rightarrow R_1$					29,418
2	$P_2 \rightarrow R_1$		+		✓	37,678
3	$R_1 \rightarrow P_2$			+	✓	56,627
4	$R_1 \rightarrow 2R_1$			+	✓	57,162
5	$R_1 + P_2 \rightarrow \emptyset$		+	+	✓	11,310
6	$R_1 \rightarrow \emptyset$			+	✓	56,781
7	$P_1 \rightarrow \emptyset$					29,410
8	$P_2 \rightarrow \emptyset$		+		✓	37,800
9	$\emptyset \rightarrow P_1$, $P_2 \rightarrow P_1 + P_2$		+		✓	58,089
10	$\emptyset \rightarrow P_2$, $P_1 \rightarrow P_1 + P_2$ (reference)					29,531

Table S4J. Multinomial logistic regression model fitting summary table for the Heat Shock Response model for Case 2b using 20 trajectories.

Reactant:Reaction	Estimate	Std. Error	Z-score
P_1:1	-0.0177	0.0306	-0.578
P_1:2	-1.0393	0.0264	-39.358***
P_1:3	-1.0023	0.0251	-39.991***
P_1:4	-1.0183	0.0249	-40.964***
P_1:5	-0.9909	0.0377	-26.257***
P_1:6	-1.0033	0.0250	-40.088***
P_1:7	-0.0155	0.0306	-0.505
P_1:8	-0.9915	0.0273	-36.275***
P_1:9	-0.9935	0.0253	-39.241***
P_2:1	0.0164	0.0327	0.501
P_2:2	1.0237	0.0311	**32.968*****
P_2:3	0.0327	0.0282	1.157
P_2:4	0.0464	0.0281	1.650
P_2:5	1.0481	0.0458	**22.862*****
P_2:6	-0.0060	0.0282	-0.212
P_2:7	0.0514	0.0328	1.569
P_2:8	1.0009	0.0313	**31.969*****
P_2:9	0.6336	0.0284	**22.318*****
R_1:1	0.0181	0.0275	0.660
R_1:2	0.0094	0.0260	0.361
R_1:3	0.9768	0.0247	**39.627*****
R_1:4	1.0014	0.0246	**40.661*****
R_1:5	0.9805	0.0396	**24.769*****
R_1:6	1.0257	0.0247	**41.566*****
R_1:7	0.0123	0.0275	0.447
R_1:8	0.0147	0.0260	0.565
R_1:9	0.0226	0.0239	0.945

The intercept terms are omitted. * < .05, ** < .01, and *** < .001

Table S4K. Species identification in the SIR model with demography using logistic regression. Symbol "+" indicates that estimated coefficients are significant and have positive signs. Symbol ✓ indicates correct identification of the corresponding reaction.

No.	Reactions	S	I	R		# of Obs.
1	$S + I \to 2I$	+	+		✓	3,248
2	$I \to R$		+		✓	3,105
3	$S \to \emptyset$	+			✓	60
4	$I \to \emptyset$		+		✓	15
5	$R \to \emptyset$			+	✓	174
6	$\emptyset \to S$ (reference)					3,033

Table S4L. Multinomial logistic regression model fitting summary table for the SIR model.

Reactant:Reaction	Estimate	Std. Error	Z-value
S:1	0.8344	0.0673	**12.398*****
S:2	-0.1268	0.0667	−1.901
S:3	1.4994	0.3769	**3.978*****
S:4	0.3065	0.7062	0.434
S:5	-0.2720	0.2242	−1.213
I:1	0.9601	0.0399	**24.035*****
I:2	1.0298	0.0426	**24.193*****
I:3	-0.0083	0.0204	−0.408
I:4	1.8572	0.5762	**3.223*****
I:5	-0.0094	0.0164	−0.576
R:1	-0.0112	0.0091	−1.225
R:2	0.0038	0.0131	0.291
R:3	-0.0430	0.0184	−2.337*
R:4	0.1112	0.3528	0.315
R:5	0.8233	0.1661	**4.958*****

The intercept terms are omitted. $^{*} < .05$, $^{**} < .01$, and $^{***} < .001$
