# Supplementary material for: Inferring structure and parameters of stochastic reaction networks with logistic regression
Source: PLoS One. 2026 Feb 12;21(2):e0341639. doi: 10.1371/journal.pone.0341639 (PMC12900436; doi:10.1371/journal.pone.0341639)
Supplement: S5 Supplement — Additional calculations and results supporting the paper’s conclusions. (PDF) [file pone.0341639.s005.pdf]

Inferring structure and parameters of stochastic reaction networks with logistic regression

Boseung Choi[1,2,3†], Hye-Won Kang[4†], Grzegorz A. Rempala[3†*],

1 Korea University Sejong Campus, Sejong, South Korea
2 Institute for Basic Science, Daejeon, South Korea
3 The Ohio State University, Columbus, OH, USA
4 University of Maryland, Baltimore County, Baltimore MD, USA

†These authors contributed equally to this work.
* rempala.3@osu.edu

S5 Supplement: Additional Model Details

The following supplemental sections provide technical details that support, but are not required for, the main presentation. In particular, we include (i) a derivation showing that the embedded Markov chain likelihood for a certain class of mass–action reaction network is equivalent to the likelihood of a multinomial logistic regression model, and (ii) additional details on stochastic epidemic model with demographic turnover. Part (i) provides a theoretical justification for the usage of formula (2) in the paper.

Multinomial logistic representation

Consider a continuous-time Markov jump process $X(t) \in \mathbb{Z}^k_{\geq 0}$ generated by a reaction network with reactions $r = 0, 1, \ldots, m$, where each reaction r has a unique stoichiometric vector and a mass–action propensity of the form

$$a_r(x; \theta_r) = \theta_r \, h_r(x),$$

with scalar rate parameter $\theta_r > 0$ and mass–action factor $h_r(x)$ given by

$$h_r(x) = \begin{cases} 1, & \text{zeroth order,} \\ x_i, & \text{first order in species } i, \\ x_i x_j, & \text{second order } (i \neq j), \\ \dfrac{x_i(x_i - 1)}{2}, & \text{second order } (i = j). \end{cases}$$

Assume the inflow reaction $0 \to A$ is present and index it by $r = 0$, so $h_0(x) \equiv 1$. Assume the full sequence of jumps is observed, yielding pairs $\{(x^{(n)}, r_n)\}_{n=1}^N$. Let $N_r(x)$ denote the number of observed firings of reaction r from state x. The embedded Markov chain likelihood is

$$L_{\mathrm{EMC}}(\theta) = \prod_x \prod_{r=0}^m \pi_r(x; \theta)^{\,N_r(x)}, \qquad \pi_r(x; \theta) = \frac{a_r(x; \theta)}{\sum_{s=0}^m a_s(x; \theta)}.$$

The result below connects the embedded chain likelihood with multinomial logistic regression (MLR) and provides a formal justification of the equation (2) in the main text. Note that in our current notation we have

$$\log \frac{\pi_r(x;\theta)}{\pi_0(x;\theta)} \;=\; \log \frac{P(Y_x = r)}{P(Y_x = 0)},$$

where $Y_x \in \{0, \dots, m\}$ indicates the reaction type observed at a jump from state x.

Theorem. (*Embedded Chain as MLR*) There exist a feature map $z : \mathbb{Z}^k_{\geq 0} \to \mathbb{R}^p$ and parameter vectors (α_r, β_r) with $\alpha_0 = 0$ and $\beta_0 = 0$ such that for all x and $r \geq 0$,

$$\log \frac{\pi_r(x;\theta)}{\pi_0(x;\theta)} = \alpha_r + \beta_r^\top z(x).$$

Furthermore, $L_{\mathrm{EMC}}(\theta)$ is equal, up to a multiplicative factor independent of θ, to the multinomial logistic regression likelihood with responses r_n and covariates $z(x^{(n)})$.

Proof. Since $h_0(x) \equiv 1$,

$$\log \frac{\pi_r(x;\theta)}{\pi_0(x;\theta)} = (\log \theta_r - \log \theta_0) + \log h_r(x).$$

Define transformed covariates:

$$z_i(x) = \log x_i, \qquad i = 1, \dots, k,$$

and for homo–second–order reactions optionally

$$z_{ii}(x) = \log\left(\frac{x_i(x_i - 1)}{2}\right).$$

Each reaction's mass-action factor satisfies

$$\log h_r(x) = b_r + \beta_r^\top z(x),$$

where β_r is sparse with entries in $\{0, 1, 2\}$ recording reactant stoichiometry, and b_r collects constants such as $-\log 2$.

Set

$$\alpha_r = (\log \theta_r - \log \theta_0) + b_r, \qquad r \geq 1.$$

Then

$$\log \frac{\pi_r(x;\theta)}{\pi_0(x;\theta)} = \alpha_r + \beta_r^\top z(x),$$

giving the multinomial logistic form. Finally, let $N(x) = \sum_{r=0}^{m} N_r(x)$ be the total number of reaction events observed while the system was in state x (i.e., the total count of all reactions leaving state x). The MLR likelihood $L_{\mathrm{LREG}}(\theta)$ satisfies then:

$$L_{\mathrm{LREG}}(\theta) = \prod_x \frac{N(x)!}{\prod_r N_r(x)!} \cdot \prod_x \prod_{r=0}^{m} \pi_r(x;\theta)^{N_r(x)} \propto L_{\mathrm{EMC}}(\theta),$$

since the multinomial factors $\frac{N(x)!}{\prod_r N_r(x)!}$ do not depend on θ. □

Remarks. Note:

- Only the rate ratios θ_r/θ_0 are identified through the embedded chain. The overall intensity scale requires the waiting-time distribution.
- Estimation of (α_r, β_r) may be performed by ordinary multinomial logistic regression; the kinetic parameters θ_r are recovered by back-substitution.
- (*Higher–Order Reactions*) The argument extends to reactions of arbitrary finite order. If reaction r consumes $\nu_{ri} \geq 0$ molecules of species i, then

$$\log h_r(x) = \sum_{i=1}^{k} \sum_{j=0}^{\nu_{ri}-1} \log(x_i - j),$$

which is linear in the augmented covariates

$$z_{i,j}(x) = \log(x_i - j), \qquad j = 0, \ldots, \nu_{\max,i} - 1,$$

with the understanding that if $\nu_{\max,i} = 0$ for a given species i, then no such terms are included for that species. With this enlarged feature map, $\log h_r(x) = b_r + \beta_r^\top z(x)$ still holds, and hence the multinomial logistic representation remains exact for all reaction orders.

Partial observability and offset terms

In many practical settings, only a subset of the chemical species in the reaction network is observable. Let $\mathcal{O}$ denote the set of observed species and $\mathcal{U}$ the set of unobserved species. For each reaction r, the mass-action propensity takes the form

$$a_r(x; \theta_r) = \theta_r \, h_r(x_{\mathcal{O}}, x_{\mathcal{U}}),$$

where $x_{\mathcal{O}}$ and $x_{\mathcal{U}}$ denote the molecular count vectors of observed and unobserved species, respectively. When all species are observed, $h_r(\cdot)$ admits the multinomial logistic representation described in the previous subsection. When some species are unobserved, we write

$$\log h_r(x_{\mathcal{O}}, x_{\mathcal{U}}) = b_r + \beta_r^\top z(x_{\mathcal{O}}) + \gamma_r^\top z(x_{\mathcal{U}}),$$

where $z(\cdot)$ is the feature map introduced earlier. Since $x_{\mathcal{U}}$ is unavailable, the term

$$\gamma_r^\top z(x_{\mathcal{U}})$$

cannot be evaluated directly. Instead, we approximate this term by an *offset*, denoted offset_{rj}, based on auxiliary dynamical information, such as estimates obtained from a reduced-order model or filtering procedure for the unobserved subsystem at time j.

Substituting this into the embedded–chain softmax representation yields

$$\log \frac{P(Y_j = r \mid X_{\mathcal{O},j} = x_{\mathcal{O},j})}{P(Y_j = r_0 \mid X_{\mathcal{O},j} = x_{\mathcal{O},j})} = \alpha_r + \beta_r^\top z(x_{\mathcal{O},j}) + \text{offset}_{rj},$$

where r_0 denotes the chosen reference reaction (not necessarily of zeroth order), $Y_j \in \{0, \ldots, m\}$ is the reaction indicator at the jth jump, and $x_{\mathcal{O},j}$ is the observed component of the pre-jump state. In this sense, the fully observed case treated earlier corresponds to the special case $\mathcal{U} = \varnothing$, for which all offsets vanish. The example in the paper illustrates this construction in the context of a stochastic SIR model with demographic turnover.

Stochastic SIR model with demography

Let $Z_i(t)$ for $i = 1, \dots, 6$ be independent unit-rate Poisson processes. The evolution of the susceptible $S(t)$, infected $I(t)$, and recovered $R(t)$ populations is given by (see, for instance, [15])

$$\begin{aligned}
S(t) &= S(0) + Z_1(\mu n_0 t) - Z_2\left(\int_0^t \frac{\beta}{n_0} S(s)I(s)\,\mathrm{d}s\right) - Z_4\left(\int_0^t \nu S(s)\,\mathrm{d}s\right), \\
I(t) &= I(0) + Z_2\left(\int_0^t \frac{\beta}{n_0} S(s)I(s)\,\mathrm{d}s\right) - Z_3\left(\int_0^t \gamma I(s)\,\mathrm{d}s\right) - Z_5\left(\int_0^t \nu I(s)\,\mathrm{d}s\right), \\
R(t) &= R(0) + Z_3\left(\int_0^t \gamma I(s)\,\mathrm{d}s\right) - Z_6\left(\int_0^t \nu R(s)\,\mathrm{d}s\right),
\end{aligned}$$

$$S(0) = n_0, \quad I(0) = \rho n_0, \quad R(0) = 0,$$

where n_0 denotes the initial total number of susceptible individuals. Assume that n_0 is large and consider the limit as n_0 goes to infinity. The resulting mean field limiting ODE system is then given by (13).

Epidemic size and inclusion probability

In the mean field limit we need to calculate the average count of Y_2, so the formula for the unscaled epidemic size at time t is given as:

$$\tilde{\tau}_t = 1 - s_t + \mu t - \int_0^t \nu s_u \mathrm{d}u.$$

Since $\tilde{\tau}_t$ is not bounded as a function of t, we need the demography–corrected formula for the epidemic size

$$\tau_t = \frac{1 - s_t + \mu t - \int_0^t \nu s_u \mathrm{d}u}{1 + \mu t - \int_0^t \nu s_u \mathrm{d}u}, \tag{S5-1}$$

which has now the correct interpretation, under the assumptions that our t is such that the numerator above is positive (this will be always true, for instance, when $\mu > \nu$). The formula above may be also interpreted via the limiting argument as follows. The initial amount of S is n_0, but by time t there has been some changes to the initial population not due to infections (flow in and flow out of S) therefore the correct relative count should be

$$\mathcal{T} = \frac{Z_2\left(\int_0^t \frac{\beta}{n_0} S(s)I(s)\,\mathrm{d}s\right)}{S(0) + Z_1(\mu n_0 t) - Z_4\left(\int_0^t \nu S(s)\,\mathrm{d}s\right)}.$$

Now τ_t is simply the approximation to $\mathcal{T}$ for large n_0.

Synthetic data generation for Seoul COVID-19 epidemic

This algorithm generates an anonymized version of a dataset containing symptom onset and confirmation dates. It is nonparametric and preserves the empirical delay distribution while ensuring that individual-level data are not directly retained.

The observation times $\tilde{T} = (t_1, t_2, \cdots, t_l)$ can be divided into two time points: One is a time of onset (infection) and the other is a time of confirmation (recovery). Let t^k_{onset} and $t^k_{confirm}$ represent these two time point of individual k. We can also define the delay from onset to confirmation

$$d^{(k)} = t^{(k)}_{confirm} - t^{(k)}_{onset}$$

The algorithm for generating synthetic data is outlined in the following step.

Algorithm S5A Synthetic data generation for COVID-19

1: **Estimate empirical delay distribution**
Compute the empirical distribution of delays:

$$d^{(1)}, d^{(2)}, \ldots, d^{(n)}.$$

2: **Deform onset dates**
Perturb each onset date by adding discrete noise:

$$\tilde{t}^{(k)}_{onset} = t^{(k)}_{onset} + \varepsilon^{(k)}, \qquad \varepsilon^{(k)} \sim Uniform(-1, 1).$$

3: **Resample delays**
For each individual k, sample a delay $\tilde{d}^{(k)}$ from the empirical distribution:

$$\tilde{d}^{(k)} \sim \{d^{(j)}\}^n_{j=1}.$$

4: **Recalculate confirmation dates**
Construct the synthetic confirmation date as:

$$\tilde{t}^{(k)}_{confirm} = \tilde{t}^{(k)}_{onset} + \tilde{d}^{(k)}.$$

The synthetic dataset consists of the pairs $(\tilde{t}^k_{onset}, \tilde{t}^k_{confirm})$, which preserve the marginal and joint timing structure without exposing individual data directly.
