# Supplementary material for: Inferring structure and parameters of stochastic reaction networks with logistic regression
Source: PLoS One. 2026 Feb 12;21(2):e0341639. doi: 10.1371/journal.pone.0341639 (PMC12900436; doi:10.1371/journal.pone.0341639)
Supplement: Attachment — Submitted filename: Review report.pdf [file pone.0341639.s006.pdf]

In this paper, the authors focus on identifying network structure and parameters in chemical reaction networks. Through stochastic models, it demonstrates that logistic regression can recover full network structures and applies the approach to estimate SIR model parameters for the COVID-19 outbreak in Greater Seoul. In addition, by combining Bayesian regression with differential equation models, the method enables robust inference from partially observed time-series data.

Overall, the paper presents interesting and novel. I recommend that the manuscript be considered for publication after minor revisions. Below, I provide a list of several comments for the authors' consideration.

- Highlight (in italics/bold) each key concept when it is first introduced (e.g., *Togashi-Kaneko* (TK) model).

- On page 4, lines 109–114 repeat the content from lines 103–108.

- On page 5, lines 129–136, please clarify what the estimated regression coefficients represent. If possible, add one sentence explaining how to interpret the sign of a coefficient from Equation (2).

- On pages 5-6, the range of *r, j k* are missing in Equations (2), (3) and (4).

- On page 7, Figure 1 is missing. A similar situation happens on Figure 2 on page 10, etc.

- On page 7, please explain how the reaction rates in a reaction network works in Equations (6) and (7).

- On page 7, lines 203-204, please clarify the distinction between the symmetric reaction case and the asymmetric reaction case. I suggest itemizing these two scenarios or at least highlighting them.

- On page 11, line 326, please explain what each rate in the SIR model represents, similar to the clarification provided on page 15, lines 416–417.

- On pages 14-15, please explain what S_u represents in Equation (8), and clearly define all parameters appearing in Equation (12).
