# Supplementary material for: Inferring structure and parameters of stochastic reaction networks with logistic regression
Source: PLoS One. 2026 Feb 12;21(2):e0341639. doi: 10.1371/journal.pone.0341639 (PMC12900436; doi:10.1371/journal.pone.0341639)
Supplement: Attachment — Submitted filename: report.pdf [file pone.0341639.s007.pdf]

1 Summary

The paper is about the inference of the structure of a reaction network from fully observed data. Since the authors assume that full observations are available, then the stoichiometry of the network is known, unless some of the reaction vectors correspond to reactions that never occur in the observation window. However for the same stoichiometric changes different reactions may have been the cause of the jump, since catalysts may be present. Among a reaction network constituted by m reactions which possibly includes diverse reactions that causes the same stoichiometric changes, they want to select those that are really driving the system from those that does not appear to be firing. Finally they also want to estimate the parameters of the active reactions.

2 Comments

The adopted methodology is interesting in its general idea, but weird in some technical aspect. Let me be more specific. Let us fucus on a 2-d system with species A and B, and stete vector (X_1, X_2).

The reaction vector $(1, 0)$ could correspond to any of the following reactions

$$\begin{gathered} \emptyset \xrightarrow{\lambda_1} \mathrm{A} \\ \mathrm{A} \xrightarrow{\lambda_2} 2\,\mathrm{A} \\ \mathrm{B} \xrightarrow{\lambda_3} \mathrm{A} + \mathrm{B} \end{gathered} \tag{1}$$

if we limit to the bimolecular ones or to others is not

$$\begin{gathered} 2\,\mathrm{A} \xrightarrow{\lambda_4} 3\,\mathrm{A} \\ \mathrm{A}^{+}\mathrm{B} \xrightarrow{\lambda_5} 2\,\mathrm{A}^{+}\mathrm{B} \\ 2\,\mathrm{B} \xrightarrow{\lambda_6} \mathrm{A} + 2\,\mathrm{B} \\ \dots \end{gathered} \tag{2}$$

Assuming mass-action kinetics the probability that a jump has $(1, 0)$ as a stochiometryc change is explicitely give by

$$p_{(1,0)} = \frac{\lambda_1 + \lambda_2 X_1 + \lambda_3 X_2}{\text{sum of all rates in the network}}$$

in the bimolecular case or

$$p'_{(1,0)} = \frac{\lambda_1 + \lambda_2 X_1 + \lambda_3 X_2 + \lambda_4 X_1(X_1 - 1) + \lambda_5 X_1 X_2 + \lambda_6 X_1 X_2(X_2 - 1)\dots}{\text{sum of all rates in the network}}$$

In a case where a constant inflow, say $\emptyset \xrightarrow{\lambda_7} \mathrm{B}$ is certainly present among the active reactions, and no other reaction sharing the same reaction vector $(0, 1)$ is present, one could define that a jump has stoichiometric change $(0, 1)$ with probability

$$p_{\text{B inflow}} = \frac{\lambda_7}{\text{sum of all rates in the network}}$$

and using such inflow reactionas a reference one would get

$$\frac{p_{(1,0)}}{p_{\text{B inflow}}} = \lambda'_1 + \lambda'_2 a + \lambda'_3 b \dots$$

where $\lambda'_i = \frac{\lambda_i}{\lambda_7}$. This (rather a special case) would suggest that a **linear** regression (with strictly positive coefficients, and, obviously, positive covariates) would be a more reasonable model, rather than a logistic one. Whereas the assumptions that the authors make on line 116, afterwards formalized as formula (2) in their paper

$$\log \frac{p_{(1,0)}}{p_{\text{B inflow}}} = \kappa_1 + \kappa_2 a + \kappa_3 b \dots$$

that the log-ratio of the probability of occurrence of the k-th reaction relative to a reference reaction is a linear function of the molecular counts of the species is certainly wrong, and they do not even argue that it might be a resonable approximation. The assumption (2) is not at all justified.

Despite the method in (2) can be extended to more complex reaction rates as in (2), the assumption that a reference reaction with a constant input needs to be present, whereas no other reaction with the same stoichiometric change can be there is very restrictive. In the general case, where the reference reaction would also have a more complicated rate function, the model would become more complicated.

A last comment to be made is that the authors apparently do not use any information about the waiting times in each state but focus on the embedded chain alone. This reduces the amount of information in the data. Adressing this issue would require to rethink the whole paper and maybe can be seen as a second stage, for another paper.

3 Reccommendation

The paper in its current form cannot be accepted. To be considered for publication one, the authors must take one of the following actions

- either the author adopt some more reasonal assumption like those impled by model (2) (or some reasonable modification of it)
- or if they really want to stick to model (2), they have to give a rationale for it, explaing why this **seemingly wrong** assumption may for some reason turn to be *not too wrong*, or a reasonable approximation of the reality.
- provide a valid link to the code repository for reproducibility and review (currently they mention a Github without providing access details, if I'm not wrong)

Similar actions are needed to fix the Sction on Parameter Estimation.pen
