# Supplementary material for: Inferring structure and parameters of stochastic reaction networks with logistic regression
Source: PLoS One. 2026 Feb 12;21(2):e0341639. doi: 10.1371/journal.pone.0341639 (PMC12900436; doi:10.1371/journal.pone.0341639)
Supplement: Attachment — Submitted filename: responses2.pdf [file pone.0341639.s008.pdf]

Response to Reviewers Comments on the Manuscript "Inference of stochastic reaction networks via logistic regression"

December 6, 2025

Dear Editors:

We would like to thank the editors and the reviewers for their thoughtful and constructive comments on our paper. Below, we provide a point-by-point response to all comments, detailing the changes made to the manuscript. In addition to the revisions described below, we have updated the references, added clarifying explanations, and corrected various typographical and notational inconsistencies throughout the paper. We have also expanded the Supplemental Materials section. A marked version of the manuscript, with all changes clearly visible, is included with the revision.

Associate Editor

- *Besides the points highlighted by the reviewers, I suggest to better discuss the scalability of the proposed method.*

  We have added a discussion of scalability in the revised Discussion section.

Reviewer One

- *Highlight (in italics/bold) each key concept when it is first introduced (e.g., Togashi–Kaneko (TK) model).*

In the revision, we have followed this suggestion throughout the paper by emphasizing key concepts (e.g., TK model, SIR model, MCMC) when they first appear.

- *On page 4, lines 109–114 repeat the content from lines 103–108.*

  We thank the review for identifying this error. We deleted the corresponding repeated part.

- *On page 5, lines 129–136, please clarify what the estimated regression coefficients represent. If possible, add one sentence explaining how to interpret the sign of a coefficient from Equation (2).*

  Corrected as suggested. We have now specified which regression coefficients we referred to and added an explanation about a negative sign of the coefficients to the existing explanation.

- *On pages 5-6, the range of r, j k are missing in Equations (2), (3) and (4).*

  Corrected as suggested. In (2), the range of k is now provided. The symbol r represents the chosen reference reaction as explained right after (2). It is now also discussed on line 104. In (3) and (4), we added the range of j and k.

- *On page 7, Figure 1 is missing. A similar situation happens on Figure 2 on page 10, etc.*

  As per the journal requirements, all figures have been placed on separate pages at the end of the manuscript.

- *On page 7, please explain how the reaction rates in a reaction network works in Equations (6) and (7).*

  As suggested, we have added explanations of how the reaction rates in equations (6) and (7) are constructed. We have also expanded the description of the model and its interpretation. These explanations are placed directly below the corresponding equations and clearly marked.

- On page 7, lines 203-204, please clarify the distinction between the symmetric reaction case and the asymmetric reaction case. I suggest itemizing these two scenarios or at least highlighting them.

As suggested, we have clarified the distinction between the symmetric and asymmetric reaction cases, and we have italicized the corresponding terms to highlight them.

- On page 11, line 326, please explain what each rate in the SIR model represents, similar to the clarification provided on page 15, lines 416–417.

  We added explanation of each parameter as suggested, below Equation (8).

- On pages 14-15, please explain what S_u represents in Equation (8), and clearly define all parameters appearing in Equation (12).

  We added the corresponding explanations below Equation (9) (previous equation (8)) and Equation (13) (previously (12)).

Reviewer Two

- *The adopted methodology is interesting in its general idea, but weird in some technical aspect. Let me be more specific (..).*

  The example provided by the reviewer corresponds to a setting with a "many-to-one" correspondence between reaction types and stoichiometric vectors. In general, such models are considerably more challenging than those considered in our paper. Our work focuses on a simpler, but still instructive, case in which the correspondence is strictly "one-to-one." We have now made this distinction more explicit throughout the manuscript.

  When the reviewer questions the appropriateness of equation (2) in the original manuscript, their concern is well taken. The relationship assumes that the original counts X can be viewed through a monotone transformation of $\log(X)$; on this logarithmic scale, the equation reflects an exact connection between reaction rates and jump probabilities in certain cases. Motivated by the reviewers' questions—which highlighted the lack of clarity in our original exposition—we have substantially revised the manuscript to make this point clearer. We now provide more explicit explanations, both in the main text and in the Supplemental Information, regarding the motivation for the model and the role of the logarithmic transformation in equations (2) and (3). A

detailed argument establishing the validity of these equations under explicit assumptions is now included in the subsections *"Multinomial logistic representation"* and *"Partial observability and offset terms"* of the Supplemental Information.

In addition, we re-analyzed our data using the logarithmic transformation and found that the method performs more stably and efficiently than in the original formulation. We are grateful to the reviewer for this suggestion, which has significantly improved the quality of our analysis. An acknowledgment of this contribution has been added to the appropriate section of the paper. We have also expanded the Discussion section to include comments on both the "many-to-one" issue and the transformation-based perspective.

- *Despite the method in (2) can be extended to more complex reaction rates as in (2), the assumption that a reference reaction with a constant input needs to be present, whereas no other reaction with the same stoichiometric change can be there is very restrictive. In the general case, where the reference reaction would also have a more complicated rate function, the model would become more complicated.*

  We agree with the reviewer that the general conditions for identifiability of stoichiometric vectors are established in [8], and that the setting we analyze is a substantial simplification of the more general framework considered there and elsewhere in the literature. However, under our simplified assumptions, the likelihood-based method becomes scalable, as it reduces to a reweighted least-squares formulation (with an optional Bayesian extension). This computational tractability is a key advantage of the proposed approach. We now emphasize this point more clearly in the Discussion; see also our response to the Associate Editor.

- *A last comment to be made is that the authors apparently do not use any information about the waiting times in each state but focus on the embedded chain alone. This reduces the amount of information in the data. Addressing this issue would require to rethink the whole paper and maybe can be seen as a second stage, for another paper.*

  The reviewer is correct that incorporating waiting-time information would significantly expand the scope of the work and require a substantial reformulation of the methodology. This direction is indeed

beyond the scope of the current paper and could be considered in future extensions.

- *either the author adopt some more reasonable assumption like those implied by model (2) (or some reasonable modification of it)*

  As suggested, we have modified model (2) to make it more explicitly aligned with the likelihood model for the embedded discrete-time Markov chain.

- *or if they really want to stick to model (2), they have to give a rationale for it, explaining why this seemingly wrong assumption may for some reason turn to be not too wrong, or a reasonable approximation of the reality.*

  As discussed above, the earlier version of model (2) may be viewed as a monotone transformation of the logistic regression for $\log X$. We now state this more clearly in the Discussion.

- *provide a valid link to the code repository for reproducibility and review (currently they mention a Github without providing access details, if I'm not wrong) Similar actions are needed to fix the Section on Parameter Estimation.*

  We have provided the correct GitHub link as suggested and have updated the Parameter Estimation section accordingly to ensure full reproducibility.
